# Vitamin A Intake and Risk of Melanoma: A Meta-Analysis

**DOI:** 10.1371/journal.pone.0102527

**Published:** 2014-07-21

**Authors:** Yun-Ping Zhang, Rui-Xue Chu, Hui Liu

**Affiliations:** 1 Department of Dermatology, Liaocheng People's Hospital, Liaocheng, China; 2 Department of Clinical Laboratory, Liaocheng People's Hospital, Liaocheng, China; 3 Department of Gynaecology and Obstetrics, Liaocheng People's Hospital, Liaocheng, China; Van Andel Institute, United States of America

## Abstract

**Background:**

Mounting evidence from experimental and animal studies suggests that vitamin A may have a protective effect on melanoma, but the findings on the association of vitamin A intake with risk of melanoma have been inconsistently reported in epidemiologic studies. We attempted to elucidate the association by performing a meta-analysis.

**Methods:**

Eligible studies were identified by searching PubMed and EMBASE databases, as well as by reviewing the references of retrieved publications. Summary odds ratios (OR) with corresponding 95% confidence interval (CI) were computed with a random-effects model. Study-specific ORs and 95% CIs for the highest vs. lowest categories of vitamin A intake were pooled.

**Results:**

A total of 8 case-control studies and 2 prospective studies comprising 3,328 melanoma cases and 233,295 non-case subjects were included. The summary OR for the highest compared with the lowest intake of total vitamin A, retinol and beta-carotene was 0.86 (95% CI = 0.59–1.25), 0.80 (95% CI = 0.69–0.92) and 0.87 (95%CI = 0.62–1.20), respectively. Significant heterogeneity was observed among studies on vitamin A and beta-carotene intake, but not among studies on retinol intake. Subgroup and sensitivity analyses confirmed these findings. There was no indication of publication bias.

**Conclusion:**

Findings from this meta-analysis suggest that intake of retinol, rather than of total vitamin A or beta-carotene, is significantly associated with reduced risk of melanoma.

## Introduction

Malignant melanoma is a potentially fatal tumor that continues to increase in incidence, and the increase is considered real and not due to earlier detection or changes in diagnostic criteria [1], [2]. Therefore, melanoma represents a significant and growing public health burden in the U.S. and worldwide [1]. Exogenous sun exposure and several host features such as light complexions, skin reactivity to sun exposure, presence of dysplastic nevi, family history of melanoma, history of cancer, and immunosuppression have been identified as major risk factors for this malignancy[3]–[6]. An understanding of other factors, in particular behavioral factors associated with melanoma risk is however less clear. Behavioral factors are modifiable, and so it is of particular importance to study their role in the etiology of cancer.

The effect of vitamin A, an essential nutrient that cannot be synthesized by humans, on melanoma development is of particular interest. Retinoids (e.g. retinol) have been documented to be effective in inhibiting proliferation, inducing apoptosis and differentiation, as well as inhibiting growth of murine and human melanoma cell lines[7]–[9]. Dietary carotenoids with provitamin A activity (mainly beta-carotene) are of antioxidant properties, and have been reported to reduce the risk of ultraviolet light-induced skin tumors in mice[10]. Epidemiologic studies have also reported a possible association between intake of vitamin A, retinol, or beta-carotene and risk of melanoma, but the findings have been inconsistent and inconclusive [11]–[20].

To the best of our knowledge, there has been no comprehensive quantitative assessment of the association of vitamins A intake with risk of melanoma. In response, we took up this meta-analysis summarizing published case-control and cohort studies to further elucidate this issue.

## Materials and Methods

### Literature search

Potentially relevant publications were searched on PubMed and EMBASE databases through February, 2014 using the following search terms: (melanoma OR skin neoplasm OR skin cancer) AND (vitamin A OR retinol OR carotene OR provitamin A OR carotenoids) AND (cohort OR prospective OR retrospective OR nested OR case-control). In addition, the reference lists of retrieved publications were also carefully reviewed for any further studies.

### Study selection

Studies were eligible for inclusion if they met the following inclusion criteria: (1) study design was case-control or cohort; (2) exposure of interest was vitamin A intake, or intake of two selected measures of vitamin A (retinol and beta-carotene); (3) outcome of interest was melanoma; and (4) odds ratio (OR), relative risk (RR) or hazard ratio (HR) with corresponding 95% confidence interval (CI) were reported. The titles and abstracts of all potentially relevant publications were reviewed to evaluate the relevance of the information; full texts were scrutinized if any potentially relevant information was identified in a retrieved abstract. For publications with same population resources or overlapping datasets, the largest or most recent one was included.

### Data extraction

Using a standardized data-collection protocol, the following data were extracted from each study: the first author's last name, publication year, country of origin, study design, age and sex, number of cases/non-cases, exposure details, ascertainment of exposure, risk estimates with corresponding 95% CIs for the highest compared with lowest levels of vitamin A intake, and variables accounted for in the analysis. If both the crude risk estimates and multivariate-adjusted ones were reported, the one reflecting the greatest degree of adjustment was extracted. Data abstraction was performed independently by two reviewers, and any discrepancy was discussed and adjudicated by a third reviewer until a consensus was achieved.

### Statistical analysis

A random-effects model taking into account both within- and between-study variation was assigned to compute the summary risk estimates [21]. We combined the data from case-control and cohort studies, and so OR with 95% CI is the measure of effect of interest in this meta-analysis. RR and HR in cohort studies were considered as OR approximations because the risk of melanoma is sufficiently low. We pooled study-specific ORs and 95% CIs for the highest vs. lowest intake to evaluate the associations between vitamin A and risk of melanoma. When results for different sources of vitamin A were reported (total, diet and supplement), the results for total vitamin A were used in the primary analysis. Stratified analysis by study design, geographic areas, sources of vitamin A (total, diet and supplement) and adjustment for sun exposure related factors were also carried out. Sensitivity analysis by omitting 1 study at each turn and combining the results from remainder were also conducted.

Statistical heterogeneity was assessed with Q and *I* ^2^ statistics [22]. For the Q statistic, a *P*-value of less than. 1 was considered statistically significant heterogeneity. Potential publication bias was evaluated by using Egger's test and Begg's funnel plot [23]. All statistical analyses were performed using STATA software, version 12.0.

## Results

### Study characteristics

**Figure 1:**
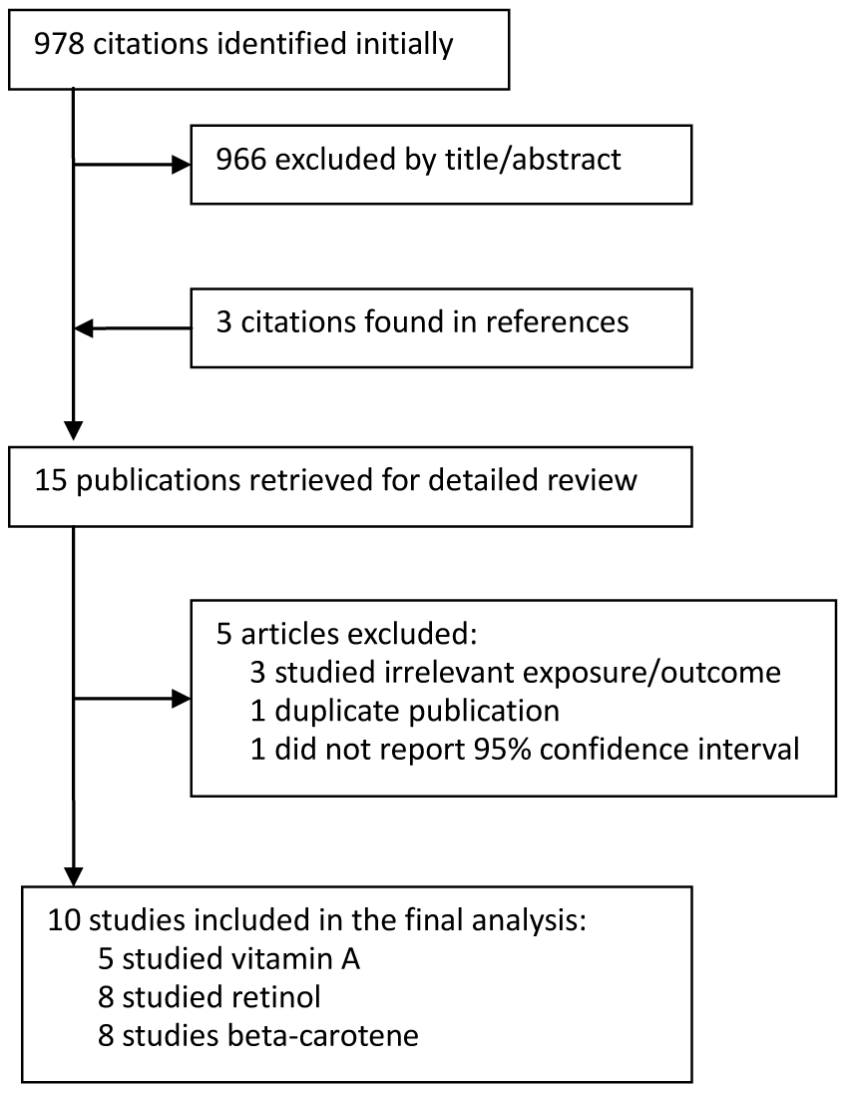
Flow diagram of systematic literature search.

The search strategy yielded 978 citations (Figure 1). Three additional citations were found in references. After carefully reading the title/abstract of these publications, 15 full texts were retrieved and assessed in more detail. After excluding publications that studied irrelevant exposure/outcome, or used duplicate populations, or failed to report 95% CI (the characteristic of the excluded studies and the reasons for exclusion are reported in Table S1), a total of 10 studies, including 8 case-control studies and 2 prospective cohort studies that met our eligibility criteria were included in the meta-analysis. These studies were published between 1986 and 2012, comprising 3,328 melanoma cases and 233,295 non-case subjects. Six studies were conducted in the United States, 2 studies were from Italy and 2 studies were carried out in Australia. The number of studies investigating the association of melanoma risk with intake of vitamin A, retinol and beta-carotene was 5, 8 and 8 respectively. Diet intake was assessed with a self-administered food-frequency questionnaire (FFQ) in 7 studies, and by interviewing participants in the remainder. The main characteristics of the included studies are reported in Table 1.

**Table 1:** Study characteristics of included case-control and cohort studies on vitamin A intake and melanoma risk.

Study(country)	Design	Age, years	Sex	Cases/controls	Exposure details	OR/RR (95%CI) (highest vs. lowest)	Diet ascertainment	Adjustment
Holman, 1986 (Australia)	PCC	N.R.(cases were matched to controls for ±5 years)	M/F	511/511	Vitamin A (S)	OR: 1.4 (0.9–2.3)	Interview	Age, sex, and electoral subdivision.
Stryker, 1990 (USA)	HCC	Cases: 44.8	M/F	204/248	Retinol (T)	OR: 0.8 (0.4–1.3)	Self-reported FFQ	Age, sex, total energy intake, hair color and ability to tan.
		Controls: 39.2						
					Retinol (D)	OR: 0.9 (0.5–1.5)		
Bain, 1993 (Australia)	PCC	Cases:49.8	F	41/297	β-carotene (D)	OR: 0.33 (0.13–0.79)	Self-reported FFQ	Age, hair color, number of painful sunburns, and education.
		Controls: 54.0						
Kirkpatrick, 1994 (USA)	PCC	Cases: 25–65	M/F	234/248	Vitamin A (T)	OR: 1.03 (0.58–1.83)	Self-reported FFQ	Age, sex, area education and total energy intake
		Controls: N.R.						
					Vitamin A (D)	OR: 1.15 (0.67–2.00)		
					Retinol (D)	OR: 1.20 (0.66–2.19)		
					β-carotene (D)	OR: 1.43 (0.80–2.54)		
Feskanich, 2003 (USA)	Cohort	25–77	F	414/161586	Retinol (T)	RR: 0.85 (0.63–1.16)	Self-reported FFQ	Age, BMI follow-up cycle, skin reaction after sun exposure, number of sunburns/moles, hair colour, family history of melanoma, residence, menopausal status, use of oral contraceptive/postmenopausal hormone, parity, height and multivitamin and vitamin A supplement use (for dietary retinol).
					Retinol (D)	RR: 1.07 (0.74–1.55)		
					β-carotene (T)	RR: 1.22 (0.86–1.74)		
Millen, 2004 (USA)	HCC	Cases:50	M/F	479/561	Retinol (D)	OR: 0.63 (0.40–0.99)	Self-reported FFQ	Age, sex, study site, confirmed dysplastic nevi status, education, and skin response after sun exposure.
		Controls: 50						
					β-carotene (T)	OR: 0.38 (0.22–0.56)		
					β-carotene (D)	OR: 0.36 (0.22–0.56)		
Naldi, 2004 (Italy)	HCC	Cases:54	M/F	542/538	Vitamin A (D)	OR: 0.51 (0.35–0.74)	Interview	Age, sex, smoking, education, BMI, history of sunburns, propensity to sunburns, number of naevi and freckles, and skin, hair and eye colour.
		Controls:54						
					Retinol (D)	OR: 0.57 (0.39–0.83)		
					β-carotene (D)	OR: 0.71 (0.50–1.02)		
Vinceti, 2005 (Italy)	PCC	Cases: 55.9	M/F	59/59	Vitamin A (D)	OR: 0.64 (0.14–2.95)	Self-reported FFQ	Age and sex
		Controls: matched to cases (±5years)						
					Retinol (D)	OR: 1.94 (0.33–11.54)		
					β-carotene (D)	OR: 1.60 (0.42–6.12)		
Le Marchand, 2006 (USA)	PCC	Case:53.7	M/F	278/278	Retinol (D)	OR: 1.1(0.5–2.5) (M) OR: 0.9 (0.4–2.7) (F)	Interview	Age, height, education, hair color, number of sunburns, ability to tan and intakes of total energy and alcohol.
		Control:52.1						
					Retinol (S)	OR: 0.6 (0.3–1.4) (M) OR: 1.8 (0.8–4.2) (F)		
					β-carotene (D)	OR: 1.0 (0.5–2.0) (M) OR: 1.1 (0.4–2.5) (F)		
Asgari, 2012 (USA)	Cohort	50–76	M/F	566/69069	Vitamin A (T)	HR: 0.87 (0.66–1.13)	Self-reported FFQ	Age, sex, education, BMI, alcohol, freckles, severe sunburns, hair colour, reaction to sunlight, family history of melanoma, history of NMSC, mole removed, macular degeneration, and energy intake (for dietary intake).
					Vitamin A (D)	HR: 1.16 (0.84–1.59)		
					Vitamin A (S)	HR: 0.88 (0.66–1.18)		
					Retinol (T)	HR: 0.84 (0.64–1.10)		
					Retinol (D)	HR: 0.85 (0.62–1.16)		
					Retinol (S)	HR: 0.74 (0.55–1.00)		
					β-carotene (T)	HR: 1.13 (0.86–1.49)		
					β-carotene (D)	HR: 1.15 (0.87–1.53)		
					β-carotene (S)	HR: 1.08 (0.86–1.36)		

BMI, body mass index; FFQ, food-frequency questionnaire; NMSC, non-melanoma skin cancer; HCC, hospital based case-control; PCC, population based case-control; N.R., not reported; M, male; F, female; T, total; D, diet; S, supplement; OR, odds ratio; RR, relative risk; CI, confidence interval.

### Vitamin A

**Figure 2:**
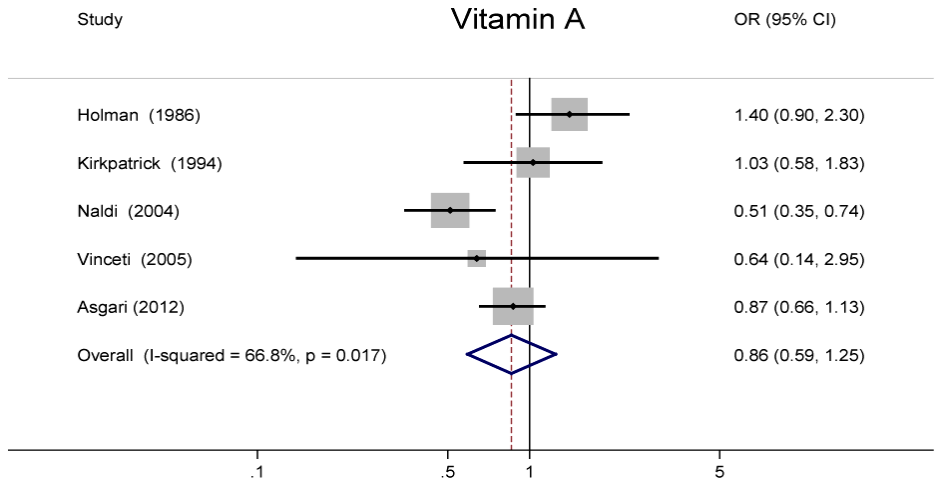
Odds ratios and 95% confidence interval of melanoma for the highest vs. lowest intake of vitamin A for individual studies and all studies combined.

Figure 2 shows OR and 95% CI of melanoma for the highest vs. lowest intake of vitamin A for each study and all studies combined. The summary OR was 0.86 (95% CI = 0.59–1.25), which suggested that vitamin A intake was not significantly associated with decreased risk of melanoma, with evidence of heterogeneity (*P* = 0.02, *I* ^2^ = 66.8%).

### Retinol

**Figure 3:**
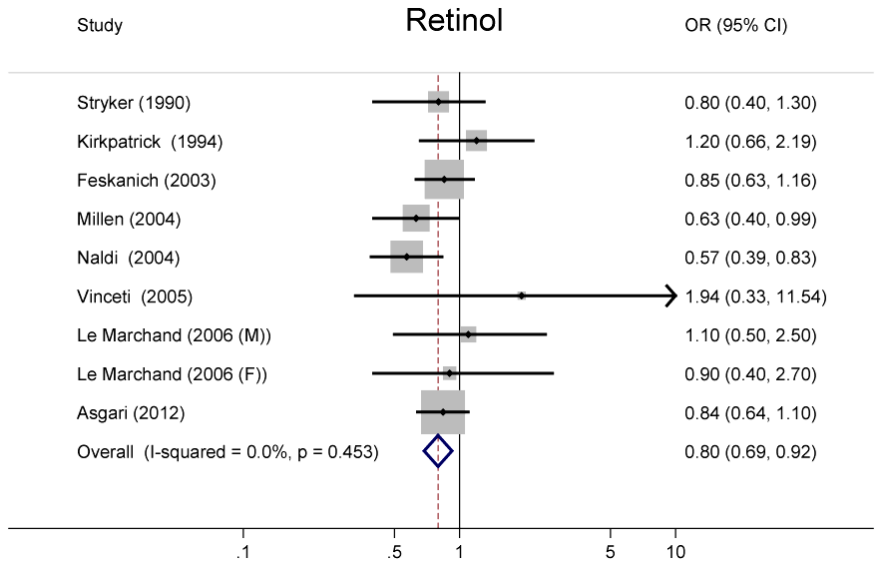
Odds ratios and 95% confidence interval of melanoma for the highest vs. lowest intake of retinol for individual studies and all studies combined.

A pooled analysis of 8 studies suggested that subjects with the highest intake of retinol had 20% lower risk of melanoma (OR = 0.80, 95% CI = 0.69–0.92), when comparing those with the lowest intake. There was no heterogeneity among studies (*P* = 0.45, *I* ^2^ = 0.0%) (Figure 3).

### Beta-carotene

**Figure 4:**
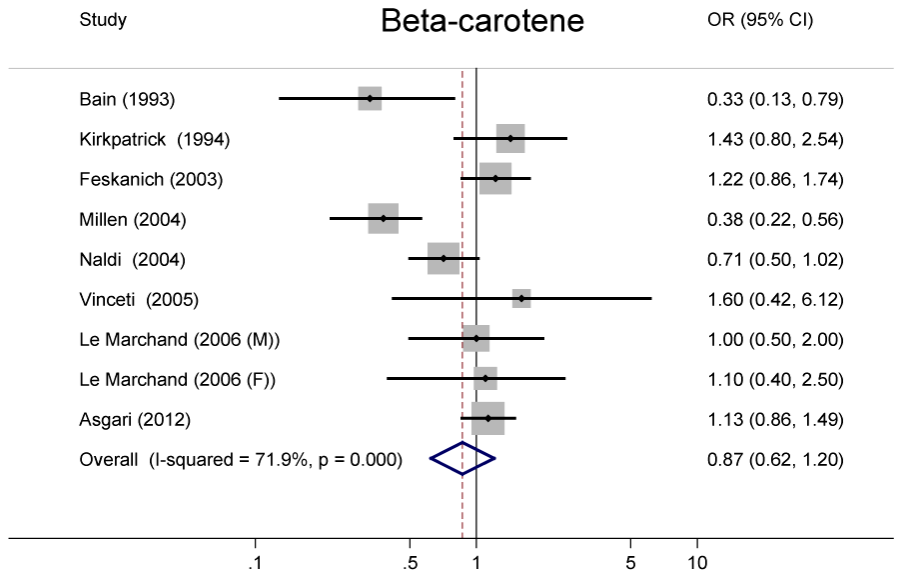
Odds ratios and 95% confidence interval of melanoma for the highest vs. lowest intake of beta-carotene for individual studies and all studies combined.

A pooled of 8 studies also found no significant inverse association between beta-carotene intake and risk of melanoma, with a summary OR of 0.87(95%CI = 0.62–1.20), with significant heterogeneity (*P*<0.001, *I* ^2^ = 71.9%) (Figure 4).

### Subgroup and sensitivity analyses

Results of subgroup analysis are reported in Table 2. Vitamin A intake was not associated with risk of melanoma in the subgroup analysis, except for a 48% reduction in risk among Italian population. This result, however, was based on only 2 studies. The observed inverse between retinol intake and risk of melanoma was consistent in the subgroup analysis, although not always of statistical significance. No significant association was found between beta-carotene intake and risk of melanoma. In addition, sensitivity analysis by excluding 1 study at each turn and pooling results from the remainder further confirmed the robust of our findings. For example, the summary OR for retinol intake ranged from 0.78 (95% CI = 0.66–0.90) to 0.84(95% CI = 0.72–0.997).

**Table 2:** Summary odds ratio of melanoma for the highest compared with lowest intake of vitamin A.

	Vitamin A				Retinol				Beta-carotene			
	*N*	OR (95% CI)	*P*	*I* ^2^ (%)	*N*	OR (95% CI)	*P*	*I* ^2^ (%)	*N*	OR (95% CI)	*P*	*I* ^2^ (%)
Design												
Case-control	4	0.86 (0.48–1.53)	0.01	74.7	6	0.77 (0.60–0.98)	0.31	15.6	6	0.76 (0.49–1.18)	0.01	68.1
Cohort	1	0.87 (0.66–1.14)	-	-	2	0.84 (0.69–1.03)	0.96	0.0	2	1.16 (0.94–1.44)	0.74	0.0
Areas												
USA	2	0.90 (0.70–1.14)	0.60	0.0	6	0.84 (0.71–0.99)	0.76	0.0	5	0.96 (0.64–1.43)	0.00	75.8
Italy	2	0.52 (0.36–0.74)	0.78	0.0	2	0.76 (0.27–2.12)	0.19	42.7	2	0.82 (0.45–1.49)	0.25	24.2
Australia	1	1.40 (0.88–2.24)	-	-	0	-	-	-	1	0.33 (0.13–0.81)	-	-
Sources of vitamin												
Total	2	0.90 (0.70–1.14)	0.60	0.0	3	0.84 (0.69–1.02)	0.98	0.0	3	0.82 (0.44–1.55)	0.00	89.4
Diet	4	0.85 (0.51–1.42)	0.01	75.2	8	0.84 (0.70–1.01)	0.27	19.7	7	0.81 (0.55–1.20)	0.00	74.2
Supplement	2	1.07 (0.68–1.67)	0.10	63.2	2	0.87 (0.51–1.47)	0.11	55.2	1	1.08 (0.86–1.36)	-	-
Adjustment for sun exposure related factors												
Yes	2	0.86 (0.66–1.12)	0.70	0.0	6	0.82 (0.70–0.98)	0.83	0.0	6	0.83 (0.54–1.27)	0.00	75.7
No	3	0.89 (0.46–1.71)	0.00	83.0	2	0.80 (0.39–1.65)	0.04	76.4	2	0.97 (0.49–1.92)	0.04	75.5

OR, odds ratio; CI, confidence interval; N, number of studies; P, p-value for heterogeneity tests.

### Publication bias

There was little indication of publication bias for studies on vitamin A, retinol or beta-carotene (*P* for Egger's test was 0.88, 0.31 and 0.69, respectively), which was supported by the visual inspection of the Begg's funnel plot.

## Discussion

To our knowledge, the present study is the first meta-analysis of the association between vitamin A and its selected measures and risk of melanoma. By analyzing the data from 10 epidemiologic studies involving more than 3,300 melanoma cases, we found that retinol intake, rather than total vitamin A or beta-carotene intake was associated reduced risk of melanoma. The subjects with the highest intake of retinol were found to have a 20% (95% CI = 8%–31%) reduction in the risk of melanoma, when comparing those with the lowest intake. These findings were confirmed by subgroup and sensitivity analyses.

Our finding of a protective effect of retinol intake on melanoma risk was supported by evidence from experimental and animal studies. Retinol belongs to a class of compounds called retinoids [24] that have been consistently reported to enhance skin repair after ultraviolet light damage [25], to reduce the incidence of ultraviolet light-induced skin tumors in mice [25], and to inhibit the growth of murine and human melanoma cells [7], [8]. Retinoids have also been shown to be effective in treating sun-induced skin lesions and reducing risk of second primary tumor in patients with prior epithelial cancer [26]. Prospective epidemiologic evidence suggests a possible interaction between retinol intake and the anatomic site of melanoma, with sun-exposed sites showing a stronger beneficial effect than sun-protected sites, suggesting that retinol may protect against melanoma by repairing sun-induced skin damage [11].

Our finding of null association between beta-carotene intake and risk of melanoma was in line with results from a recently published meta-analysis of randomized controlled trials that reported no effect of beta-carotene supplementation on risk of melanoma (RR = 0.98, 95%CI = 0.60–1.46) [27]. Noteworthy is that the vitamin A activity of beta-carotene in foods is 1/12 that of retinol. Therefore, it is possible that the levels of beta-carotene in the primary studies were still too low to exert a protection against melanoma, or that the number of cases was still too small to detect a weak protection. It was unexpected that total vitamin A intake was not associated with risk of melanoma given the beneficial effect of retinol observed in the current study. Low statistical power resulting from small number of cases may be an alternative explanation.

Individual studies are in general of low power to detect a significant association between exposure and outcome, especially when the incidence of disease was relatively low. The major strength of our study is the inclusion of large number of melanoma cases, which had largely enhanced the power of the study.

Several limitations to this meta-analysis should also be noticed. First, most included studies are retrospective case-control studies. Exposure data in these studies were collected after case diagnosis, raising a problem of recall bias. In a study of diet and melanoma, however, it is unlikely that cases had differentially recalled their diet intakes, or that cases had changed their diet habits due to the diagnosis of the disease, because little knowledge of an association between diet and melanoma exists. However, selection bias is still a concern. Second, most included studies assessed diet intake with a self-reported FFQ, which may have resulted in measurement errors and contributed to exposure misclassification. Third, although included studies provided risk estimates accounting for multi-variables, including sun exposure related factors, some unmeasured or unknown confounding cannot completely be ruled out. Subjects with high intake of retinol may also be more likely to have other health-care behaviors that impacted melanoma risk. Fourth, dose-response analysis was not carried out because only few studies [13], [16]–[18] had provided sufficient data for this analysis. Finally, publication bias is also a consideration as this meta-analysis was based on published literature. However, no indication of publication bias was found.

In summary, results from this meta-analysis indicate that intake of retinol, but not total vitamin A or beta-carotene, is significantly associated with reduced risk of melanoma. Our study solved the inconsistency of the literatures on the associations of vitamin A intake and risk of melanoma. However, the prevention of melanoma should still rely on reducing traditional risk factors for melanoma, until the benefit of retinol is confirmed in future well-designed prospective epidemiologic studies and clinical trials.

## Supporting Information

Table S1 **Excluded publications after full-text review and the reasons for exclusion.**
(DOCX)

Checklist S1 **PRISMA checklist.**
(DOC)
