# Supplementary material for: Vitamin A Intake and Risk of Melanoma: A Meta-Analysis
Source: PLoS One. 2014 Jul 21;9(7):e102527. doi: 10.1371/journal.pone.0102527 (PMC4105469; doi:10.1371/journal.pone.0102527)
Supplement: Table S1 — Excluded publications after full-text review and the reasons for exclusion. (DOCX) [file pone.0102527.s001.docx]

**Table S1.**  Excluded publications after full-text review and the reasons for exclusion.

| Study | Year | Reasons for exclusion |
| --- | --- | --- |
| Asgari et al.[[1](#_ENREF_1)] | 2009 | The population used in this article were also used in another article[[2](#_ENREF_2)] including large number of cases. |
| Middleton et al. [[3](#_ENREF_3)] | 1986 | 95% confidence interval were not reported (total vitamin A, RR=0.62 and 0.78 for the 2th and 3th teirtle, P for trend=0.38). |
| Shors et al. [[4](#_ENREF_4)] | 2001 | No data for vitamin A were reported. |
| Freedman et al.[[5](#_ENREF_5)] | 2003 | No data for vitamin A were reported. |
| Green et al.[[6](#_ENREF_6)] | 1986 | No data for vitamin A were reported. |

1. Asgari MM, Maruti SS, Kushi LH, White E (2009) Antioxidant supplementation and risk of incident melanomas: results of a large prospective cohort study. Arch Dermatol 145: 879-882.

2. Asgari MM, Brasky TM, White E (2012) Association of vitamin A and carotenoid intake with melanoma risk in a large prospective cohort. J Invest Dermatol 132: 1573-1582.

3. Middleton B, Byers T, Marshall J, Graham S (1986) Dietary vitamin A and cancer--a multisite case-control study. Nutr Cancer 8: 107-116.

4. Shors AR, Solomon C, McTiernan A, White E (2001) Melanoma risk in relation to height, weight, and exercise (United States). Cancer Causes Control 12: 599-606.

5. Freedman DM, Sigurdson A, Doody MM, Rao RS, Linet MS (2003) Risk of melanoma in relation to smoking, alcohol intake, and other factors in a large occupational cohort. Cancer Causes Control 14: 847-857.

6. Green A, Bain C, McLennan R, Siskind V (1986) Risk factors for cutaneous melanoma in Queensland. Recent Results Cancer Res 102: 76-97.
